# Methyl vinyl ketone and its analogs covalently modify PI3K and alter physiological functions by inhibiting PI3K signaling

**DOI:** 10.1016/j.jbc.2024.105679

**Published:** 2024-01-24

**Authors:** Atsushi Morimoto, Nobumasa Takasugi, Yuexuan Pan, Sho Kubota, Naoshi Dohmae, Yumi Abiko, Koji Uchida, Yoshito Kumagai, Takashi Uehara

**Affiliations:** 1 Department of Medicinal Pharmacology, Graduate School of Medicine, Dentistry and Pharmaceutical Sciences, Okayama University, Okayama, Japan; 2 Biomolecular Characterization Unit, Technology Platform Division, RIKEN Center for Sustainable Resource Science, Wako, Saitama, Japan; 3 Graduate School of Biomedical Science, Nagasaki University, Nagasaki, Japan; 4 Laboratory of Food Chemistry, Graduate School of Agricultural and Life Sciences, The University of Tokyo, Tokyo, Japan; 5 Graduate School of Pharmaceutical Sciences, Kyushu University, Fukuoka, Japan

**Keywords:** phosphatidylinositol 3-kinase (PI 3-kinase), cell signaling, chemical modification, autophagy, glucose uptake

## Abstract

Reactive carbonyl species (RCS), which are abundant in the environment and are produced *in vivo* under stress, covalently bind to nucleophilic residues such as Cys in proteins. Disruption of protein function by RCS exposure is predicted to play a role in the development of various diseases such as cancer and metabolic disorders, but most studies on RCS have been limited to simple cytotoxicity validation, leaving their target proteins and resulting physiological changes unknown. In this study, we focused on methyl vinyl ketone (MVK), which is one of the main RCS found in cigarette smoke and exhaust gas. We found that MVK suppressed PI3K–Akt signaling, which regulates processes involved in cellular homeostasis, including cell proliferation, autophagy, and glucose metabolism. Interestingly, MVK inhibits the interaction between the epidermal growth factor receptor and PI3K. Cys656 in the SH2 domain of the PI3K p85 subunit, which is the covalently binding site of MVK, is important for this interaction. Suppression of PI3K–Akt signaling by MVK reversed epidermal growth factor–induced negative regulation of autophagy and attenuated glucose uptake. Furthermore, we analyzed the effects of the 23 RCS compounds with structures similar to MVK and showed that their analogs also suppressed PI3K–Akt signaling in a manner that correlated with their similarities to MVK. Our study demonstrates the mechanism of MVK and its analogs in suppressing PI3K–Akt signaling and modulating physiological functions, providing a model for future studies analyzing environmental reactive species.

Reactive carbonyl species (RCS) are compounds with highly reactive moieties such as α,β-unsaturated carbonyl. RCS are abundant in the environment and are produced *in vivo* under stress. For example, methyl vinyl ketone (MVK), crotonaldehyde (CRA), and methacrolein are found in cigarette smoke and exhaust gases (1, 2, 3); acrylamide (AA) is found in processed foods (4); and ethyl vinyl ketone (EVK), 3-penten-2-one (PO), propionaldehyde; and transcinnamaldehyde (t-CIA) are found in food additives such as flavorings and spices (5, 6, 7, 8). In addition, acrolein (ACR) is formed *in vivo* by lipid peroxidation (9), and itaconic acid is formed abundantly as an intermediate in the tricarboxylic acid cycle under oxidative stress (10). RCS accumulate through exposure to the living environment or diet or when their production or metabolic pathways are abnormal under stress and exhibit deleterious cytotoxic effects due to their high reactivity with proteins, RNA or DNA (11). In particular, the α,β-unsaturated carbonyl group covalently binds to nucleophilic residues such as Cys, Lys, and His in proteins *via* Michael addition reactions (12). Although many compounds are classified as RCS, only a few have been shown to react with specific biological substances and induce resulting physiological changes, and many studies have been limited to general methods such as cytotoxicity assessment.

Since intracellular signaling pathways sense extracellular or intracellular stresses to alter physiological functions, we hypothesized that they are potential targets for RCS (13, 14). PI3K–protein kinase B (Akt) signaling is one of the signaling pathways involved in cell proliferation, autophagy, and glucose metabolism that maintains cellular homeostasis. Activated PI3K phosphorylates phosphatidylinositol (4,5)-bisphosphate (3, 4) (PIP2), which is present in the membrane as a lipid, to form phosphatidylinositol (3,4,5)-triphosphate (PIP3). Increasing PIP3 levels activate Akt, which then regulates downstream signaling through a kinase cascade. Activated Akt induces the activation of mammalian target of rapamycin (mTOR), which is a part of mTOR complex 1 (mTORC1), and suppresses autophagosome formation (15). Additionally, during glucose uptake, activated Akt induces membrane translocation of glucose transporters involved in glucose translocation, including GLUT1 and GLUT4 (16, 17, 18). Disruption of physiological regulation under the control of PI3K–Akt signaling destabilizes homeostasis in the body and contributes to the development of pathologies, such as growth failure and diabetes (19).

Therefore, we hypothesized that certain RCS affect PI3K–Akt signaling. However, due to the enormous amount of time required to analyze all RCS, we decided to first focus on one specific compound and then analyze RCS with similar structures. We focused on MVK, one of the RCS found in cigarette smoke and exhaust gases (3). Since, for example, cigarette smoke has been implicated in the development of pathologies such as growth failure and diabetes (20, 21) and contains a high concentration (approximately 30 μg) of MVK (22), environmental exposure to MVK is expected to significantly affect biological functions. Indeed, MVK has an α,β-unsaturated carbonyl group (Table S1) and can covalently bind to nucleophilic residues on proteins (23, 24). On the other hand, analyses of the effects of MVK exposure have been limited to studies on cytotoxicity (3, 25, 26), and the target proteins and physiological effects of MVK remain unknown.

In this study, we demonstrated that MVK negatively regulates PI3K–Akt signaling by inhibiting the interaction between receptor and PI3K and modifies physiological functions such as autophagy and glucose uptake. Interestingly, MVK covalently modified the Cys residue of the PI3K p85, which is important for the interaction between receptor tyrosine kinases (RTKs) and PI3K. In addition, we analyzed the effects of 23 RCS with structural similarity to MVK on PI3K–Akt signaling and found that several MVK analogs suppressed Akt phosphorylation in a manner correlated with their MVK similarity. Our study helps to elucidate the physiological changes caused by MVK and its structural analogs, and our model analysis system will pave the way for assessments of the toxicity of other environmental reactive species.

## Results

### MVK suppresses the PI3K–Akt pathway

**Figure 1:**
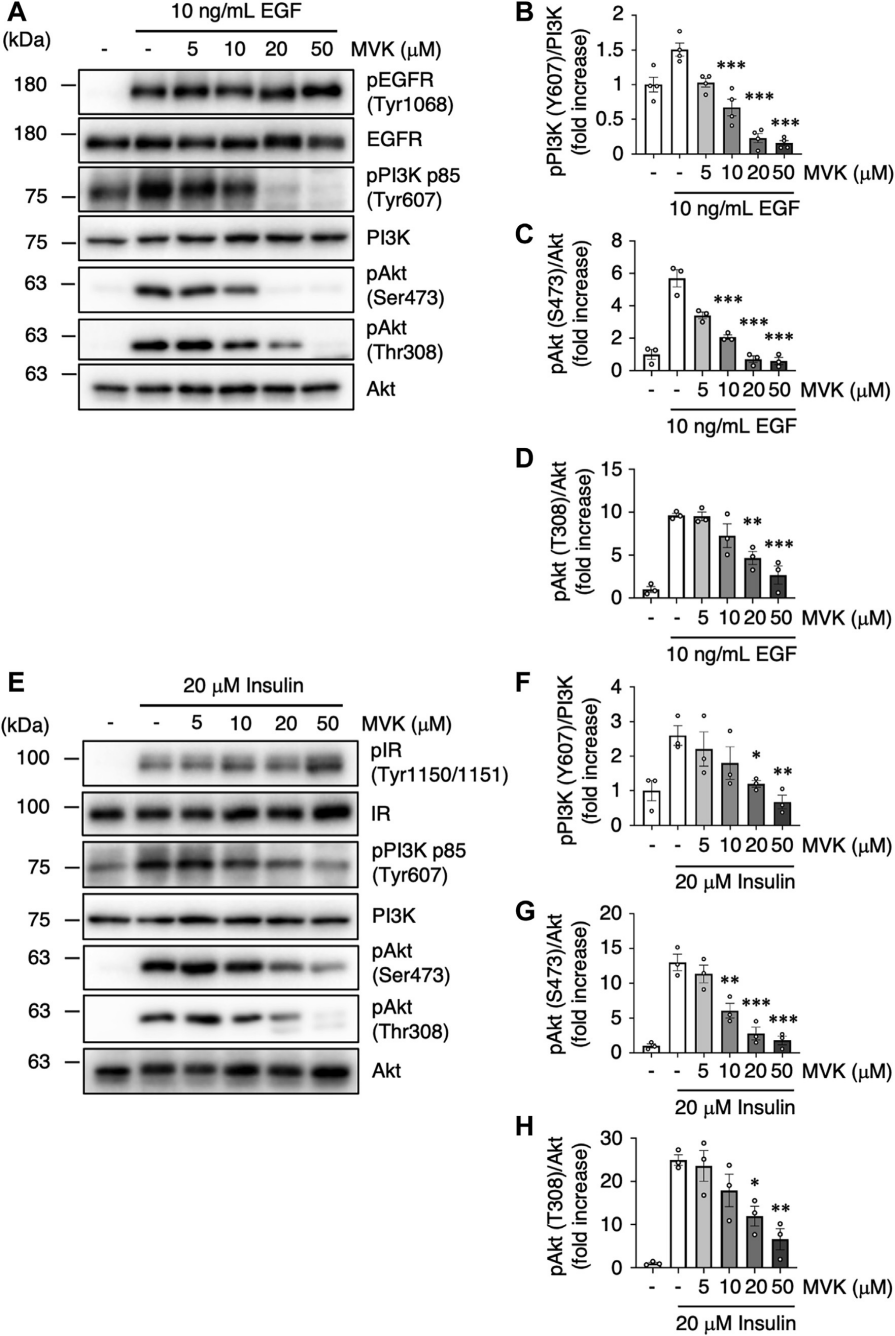
**MVK suppresses the phosphorylation of PI3K and Akt independently of RTKs.***A*–*H*, A549 cells were incubated with serum-free medium for 24 h at 37 °C. After exposure to the indicated concentrations of MVK for 30 min, cells were stimulated with 10 ng/ml EGF (*A*–*D*) or 20 μM insulin (*E*–*H*) for 10 min. The levels of phosphorylated PI3K (pPI3K), total PI3K, phosphorylated Akt (pAkt) (Ser473 and Thr308), total Akt, phosphorylated EGFR (pEGFR), total EGFR, phosphorylated IR (pIR), and total IR were evaluated by Western blotting (*A* and *E*). The levels of pPI3K (*B* and *F*), pAkt (Ser473) (*C* and *G*), and pAkt (Thr308) (*D* and *H*) were quantified and normalized to those of total PI3K and Akt. The data are the mean ± SEM., ∗*p* < 0.05, ∗∗*p* < 0.01, and ∗∗∗*p* < 0.001 *versus* EGF. Statistical analyses were performed using one-way ANOVA with Bonferroni’s multiple comparisons test. EGF, epidermal growth factor; EGFR, epidermal growth factor receptor; MVK, methyl vinyl ketone; RTK, receptor tyrosine kinase.

PI3K–Akt signaling is driven by the autophosphorylation and activation of RTKs, such as epidermal growth factor receptor (EGFR) and insulin receptor (IR), induced by epidermal growth factor (EGF) and insulin (27). Phosphorylation of the PI3K p85 subunit at Tyr607 and phosphorylation of Akt at Thr308 and Ser473 are also indicators of the activation of each of these kinases (28, 29, 30). To investigate effects of MVK on PI3K–Akt signaling, we measured the phosphorylation of PI3K and Akt in cultured cells. To exclude the effects of other growth factors in serum, the following experiments were performed with human adenocarcinoma A549 cells in serum-free medium. Interestingly, pretreatment with MVK dose dependently reduced the phosphorylation of PI3K and Akt induced by EGF treatment (Fig. 1, *A*–*D*). Additionally, we found that MVK suppressed EGF-induced phosphorylation of PI3K and Akt in MDA-MB-231 human breast adenocarcinoma cells and in human small airway epithelial cells (Fig. S1, *A* and *B*). These results suggest that MVK suppresses EGF-induced activation of PI3K and Akt independently of cell type.

**Figure 2:**
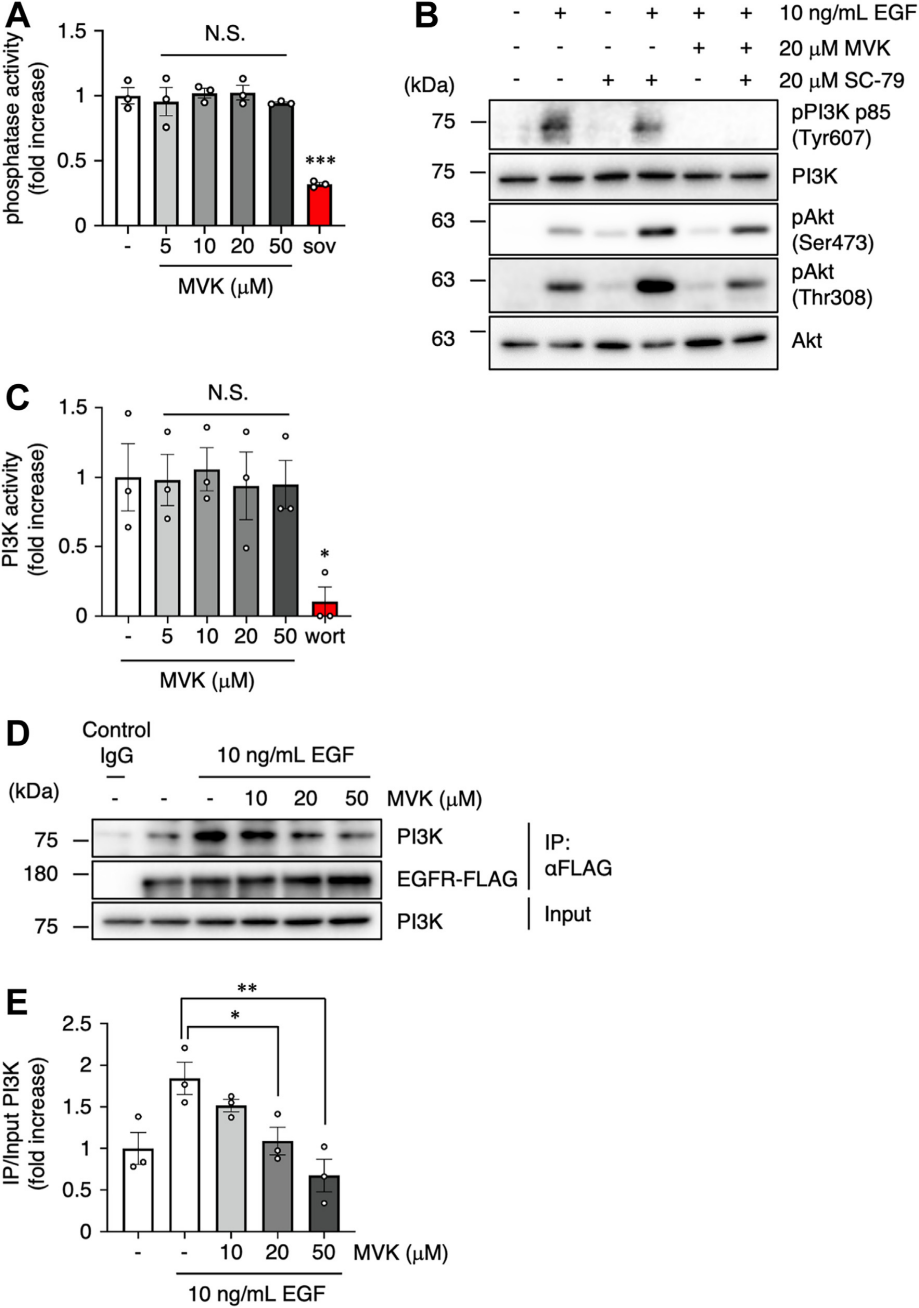
**MVK inhibits the interaction between EGFR and PI3K.***A*, A549 cells were treated with the indicated concentration of MVK for 30 min or 20 mM sodium orthovanadate (sov) for 60 min. The phosphatase activity in the cell lysates was measured using the pNPP assay. The data are the mean ± SEM, n = 3, ∗*p* < 0.05 *versus* control. Statistical analysis was performed using one-way ANOVA with Bonferroni’s multiple comparisons test. *B*, cells were cultured in serum-free medium for 24 h. After exposure to 20 μM MVK for 30 min, the cells were treated with 20 μM SC-79 for 30 min and/or 10 ng/ml EGF for 10 min. The lysates were analyzed by Western blotting with anti-pPI3K, anti-PI3K, anti-pAkt (Ser473 and Thr308), and anti-Akt antibodies. *C*, recombinant PI3K protein (20 ng/ml) was preincubated with the indicated concentration of MVK or 1 μM wort for 30 min at 37 °C. After incubation with PIP2 substrate, the concentration of PIP3 generated by PI3K kinase activity was measured by ELISA. The data are the mean ± SEM, n = 3, ∗*p* < 0.05 *versus* control. Statistical analysis was performed using one-way ANOVA with Bonferroni’s multiple comparisons test. *D*, A549 cells were transiently transfected with pIDT-SMART (C-TSC) FLAG human EGFR. The cells were incubated with serum-free medium for 42 h and exposed to the indicated concentration of MVK for 30 min. After stimulation with 10 ng/ml EGF for 3 min on ice, the lysates were analyzed by immunoprecipitation using an anti-FLAG antibody and by Western blotting with anti-PI3K and anti-FLAG antibodies. *E*, PI3K levels within the immunoprecipitation samples were quantified and normalized to the level within the total lysate (input) sample. The data are the mean ± SEM, n = 3, ∗*p* < 0.05, and ∗∗*p* < 0.01 *versus* EGF. Statistical analysis was performed using one-way ANOVA with Bonferroni’s multiple comparisons test. EGF, epidermal growth factor; EGFR, epidermal growth factor receptor; MVK, methyl vinyl ketone; PIP2, phosphatidylinositol (4,5)-bisphosphate; PIP3, phosphatidylinositol (3,4,5)-triphosphate; pNPP, *p*-nitrophenyl phosphate.

MVK also suppressed insulin-stimulated PI3K and Akt phosphorylation, suggesting a ligand-independent effect (Fig. 1, *E–H*). Furthermore, MVK had no effect on receptor autophosphorylation, at Tyr1068 of EGFR and Tyr1140/Tyr1141 of IR, which are involved in kinase activities (31, 32), suggesting that MVK acts downstream of RTKs (Figs. 1, *A* and *E*, and S1, *A* and *B*). Phosphatase activities, such as the action of protein tyrosine phosphatases on the substrate RTK and the action of PTEN on the substrate PIP3, also contribute to the regulation of PI3K–Akt signaling (33, 34). We investigated the effect of MVK on intracellular phosphatase activity in A549 cells using *p*-nitrophenyl phosphate (pNPP) substrates, which are degraded and stained upon phosphatase activation (35). While treatment with sodium orthovanadate, a phosphatase inhibitor, significantly inhibited phosphatase activity, MVK failed to affect phosphatase activity (Fig. 2*A*). This result suggests that MVK does not act on phosphatases such as protein tyrosine phosphatases and PTEN. In contrast, the inhibitory effect of MVK on Akt phosphorylation was restored when the cells were treated with SC-79, which directly binds to and activates Akt (36) (Fig. 2*B*), suggesting that MVK acts upstream of Akt. Our results predict that MVK targets PI3K, which mediates Akt activation by RTKs.

### MVK inhibits the interaction between RTKs and PI3K

Class 1A PI3K is a heterodimer consisting of two subunits, p85 and p110. P85 recognizes and binds to a specific phosphorylation site (the YXXM motif) of the RTK and serves as the regulatory subunit that recruits p110 (37), which is responsible for the kinase activity that generates PIP3 from PIP2 (38). To investigate whether MVK directly inhibits the enzymatic activity of PI3K, we analyzed the effect of MVK using an *in vitro* PI3K assay. While treatment with wortmannin (wort), a PI3K inhibitor, markedly inhibited PI3K activity, MVK failed to inhibit it (Fig. 2*C*).

Therefore, we analyzed the binding of PI3K to RTKs, one of the functions of p85, as another regulatory system of PI3K activity. To investigate the effect of MVK on EGFR–PI3K complex formation, we performed coimmunoprecipitation analysis using A549 cells transfected with EGFR–FLAG. Consistent with previous findings (39, 40), EGF induced the EGFR–PI3K interaction, but MVK inhibited the EGF-induced interaction in a dose-dependent manner (Fig. 2, *D* and *E*). Our results suggest that MVK inhibits the interaction between RTKs and PI3K by acting on the p85 subunit and suppresses signal transduction from RTKs.

### MVK covalently modifies the p85 subunit of PI3K

**Figure 3:**
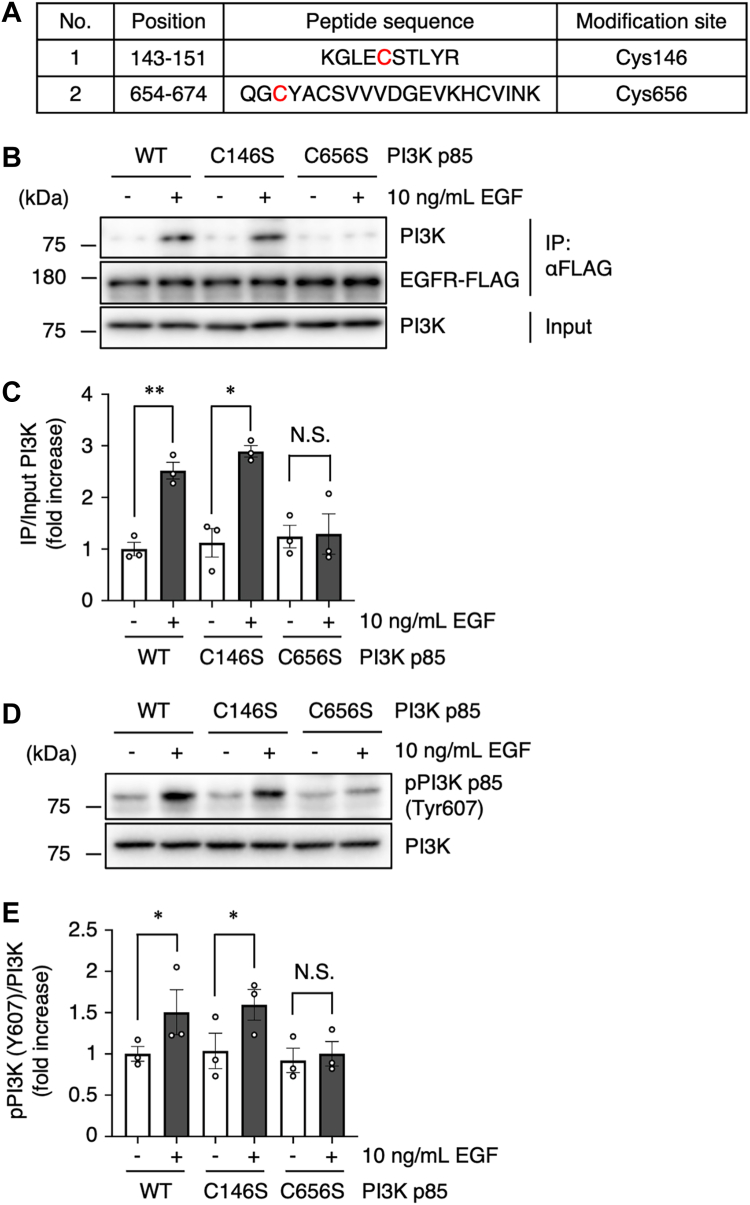
**MVK covalently modifies the p85 subunit of PI3K.***A*, identification of MVK-binding sites in recombinant PI3K p85 by LC–MS/MS. Recombinant PI3K protein (10 μg/ml) was incubated with 20 μM MVK for 10 min at RT. Trypsin-digested PI3K peptides were analyzed by LC–MS/MS. The mass data are shown in Fig. S1 and Table S2. *B* and *C*, A549 cells were transiently transfected with human EGFR-FLAG and pcDNA6 myc-His human PI3K p85 WT, C146S, or C656S. The cells were incubated with serum-free medium for 42 h and stimulated with 10 ng/ml EGF for 3 min on ice. The lysates were analyzed by immunoprecipitation with an anti-FLAG antibody and subjected to Western blotting with anti-PI3K and anti-FLAG antibodies. The data are the mean ± SEM, n = 3, ∗*p* < 0.05, and ∗∗*p* < 0.01 *versus* control. Statistical analysis was performed using one-way ANOVA with Bonferroni’s multiple comparisons test. *D* and *E*, cells were transiently transfected with WT, C146S, or C656S human PI3K p85 myc-His. The cells were incubated with serum-free medium for 42 h and stimulated with 10 ng/ml EGF for 10 min. The lysates were analyzed by Western blotting with anti-pPI3K and anti-PI3K antibodies. The data are the mean ± SEM, n = 3, ∗*p* < 0.05 *versu*s control. Statistical analysis was performed using one-way ANOVA with Bonferroni’s multiple comparisons test. EGF, epidermal growth factor; MVK, methyl vinyl ketone; RT, room temperature.

Because MVK has an α,β-unsaturated carbonyl group, MVK can modify nucleophilic protein residues such as Cys and Lys residues *via* Michael addition (23). Therefore, we hypothesized that modification of MVK is responsible for the loss of function of p85. To identify the binding site of MVK to the p85 subunit, we analyzed recombinant PI3K p85 protein exposed to MVK by LC–MS/MS. We found that MVK binds to Cys146 and Cys656 (Figs. 3*A* and S2, *A* and *B*). Cys146 is located in the Bcl-2 homology domain, which binds to small GTPases such as Rho and Rac, but the involvement of this domain in RTK binding is unknown. In contrast, Cys656 is located in the Src homology (SH2) domain at the C terminus of p85, a candidate binding site of RTKs (38, 41) (Figs. S2*C* and S3*A*). Interestingly, point mutations of residues around Cys656, such as Arg649, Lys653, and Tyr657 of p85, inhibit interactions with RTKs such as platelet-derived growth factor receptor and insulin receptor substrate 1 (42, 43), resulting in suppression of PI3K–Akt signaling. We generated p85 mutants (C146S and C656S) to test whether the identified MVK target sites are critical for binding to RTKs. We found that the EGFR–PI3K interaction was induced by EGF stimulation in the WT and the C146S mutant, but not in the C656S mutant (Fig. 3, *B* and *C*). Additionally, phosphorylation of PI3K was induced by EGF stimulation in the WT and C146S mutant, but not in the C656S mutant (Fig. 3, *D* and *E*). As the Ser hydroxy group has similar electrochemical properties but low nucleophilicity compared to those of the Cys thiol group (44), we hypothesized that MVK masks the nucleophilicity of Cys and that the CS mutants mimic the MVK binding state. These results indicate that Cys656 of p85, which is one of the target sites of MVK, is an important site for binding to RTKs to regulate PI3K activity.

### MVK alters physiological responses downstream of PI3K

**Figure 4:**
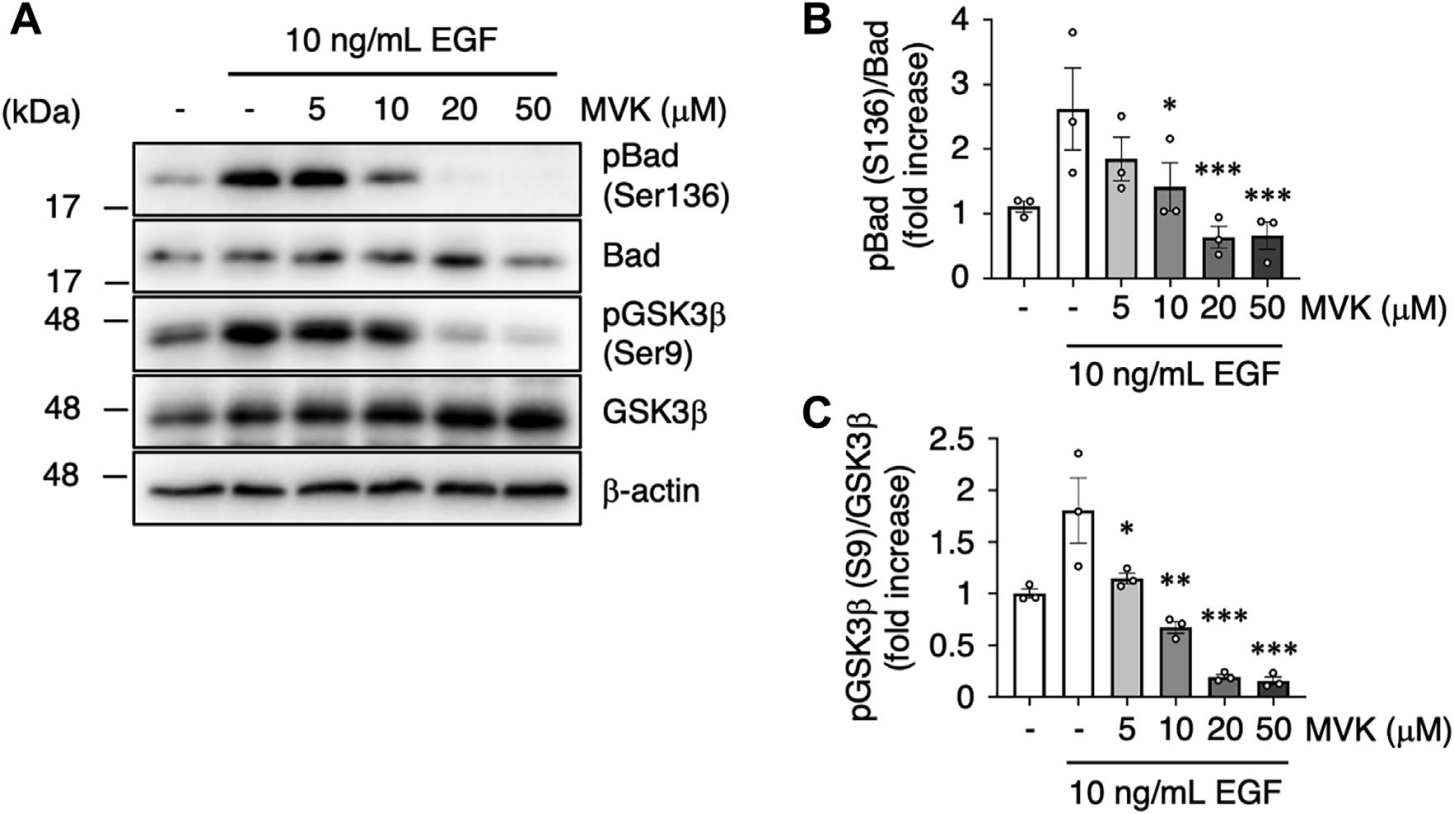
**MVK suppresses phosphorylation of Bad and GSk3β**. *A*–*C*, A549 cells were incubated with serum-free medium for 24 h and exposed to the indicated concentration of MVK for 30 min. After stimulation with 10 ng/ml EGF for 10 min, the lysates were analyzed by Western blotting with anti-phosphorylated Bad (pBad), anti-Bad, anti-phosphorylated GSK3β (pGSK3β), anti-GSK3β and anti-β-actin antibodies. The data are the mean ± SEM, n = 3, ∗*p* < 0.05, ∗∗*p* < 0.01, and ∗∗∗*p* < 0.001 *versus* EGF. Statistical analyses were performed using one-way ANOVA with Bonferroni’s multiple comparisons test. Bad, B-cell lymphoma 2–associated death promoter; EGF, epidermal growth factor; GSK3β, glycogen synthase kinase 3 beta; MVK, methyl vinyl ketone.

Next, we examined whether MVK affects downstream signaling of PI3K and physiological events. Activation of PI3K–Akt signaling phosphorylates B-cell lymphoma 2–associated death promoter (Bad), an apoptosis inducer (45) and glycogen synthase kinase 3 beta (GSK3β), a regulator of glucose metabolism (46). When we analyzed the phosphorylation of Akt target sites of Bad (Ser136) (47) and GSK3β (Ser9) (48), concomitant with MVK-mediated inhibition of PI3K and Akt phosphorylation, the phosphorylation of Bad and GSK3β were inhibited in a dose-dependent manner (Fig. 4, *A*–*C*).

**Figure 5:**
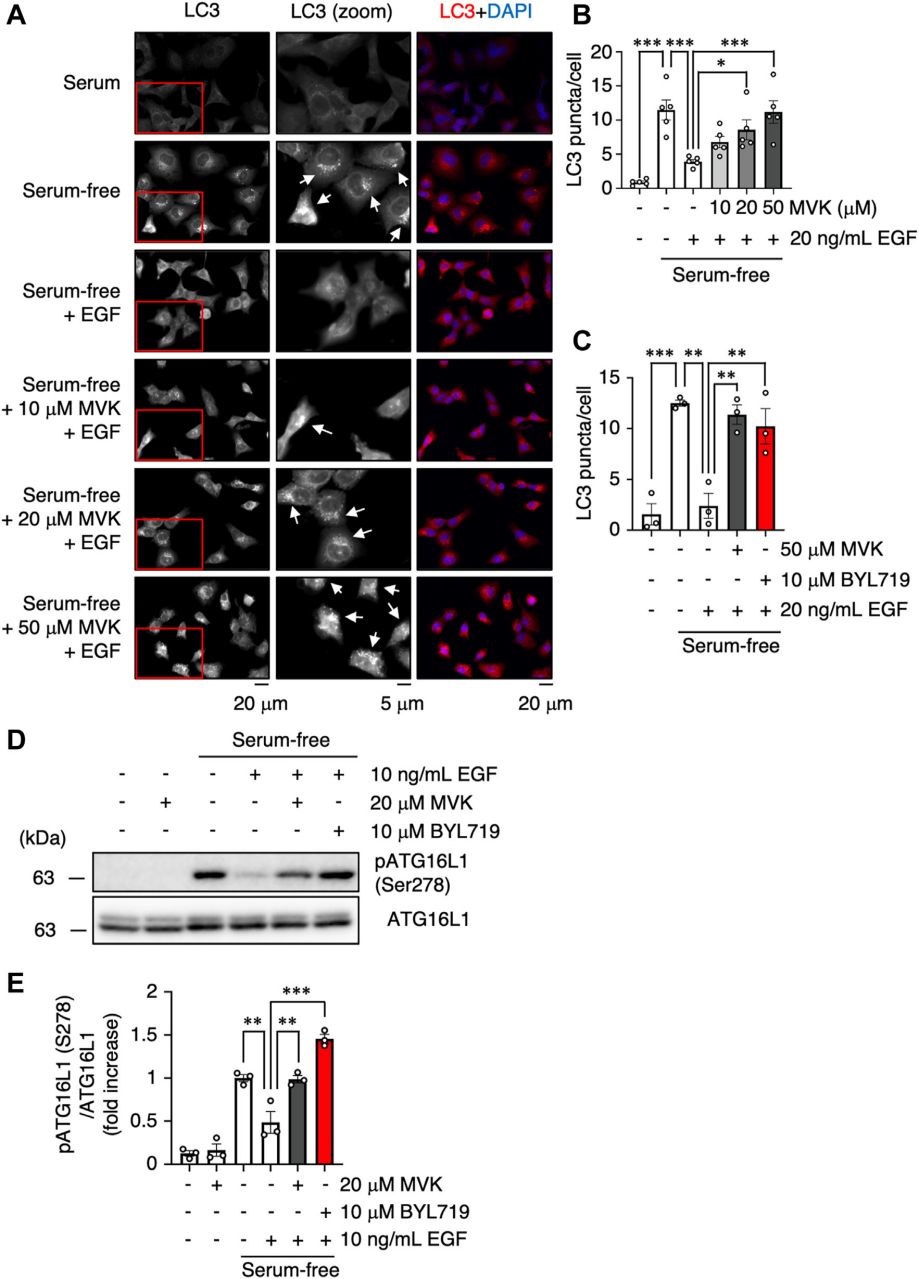
**MVK reverses EGF-induced negative regulation of autophagy**. *A*–*C*, A549 cells were incubated with serum or serum-free medium for 24 h and exposed to the indicated concentration of MVK for 30 min (*A*–*C*) or 10 μM BYL719 for 60 min (*C*). After stimulation with 20 ng/ml EGF for 1 h, the cells were immunostained for LC3 (*gray or red*) and nuclei (*blue*). The *middle column of p*anels shows magnified regions indicated by the *red fr*ame in the corresponding LC3 images. The *arrows* indicate LC3 puncta-positive cells. The scale bar represents 20 μm (*right and left columns*) or 5 μm (*middle column*). The LC3 puncta were counted using the difference of Gaussians method of ImageJ and normalized to the number of total cells. The data are the mean ± SEM, ∗*p* < 0.05, ∗∗*p* < 0.01, and ∗∗∗*p* < 0.001. Statistical analyses were performed using one-way ANOVA with Bonferroni’s multiple comparisons test. *D* and *E*, cells were incubated with serum or serum-free medium for 24 h. After pretreatment with 20 μM MVK for 30 min or 10 μM BYL719 for 60 min, the cells were stimulated with 10 ng/ml EGF for 1 h. The levels of phosphorylated ATG16L1 (pATG16L1) and total ATG16L1 were evaluated by Western blotting. The levels of pATG16L1 were quantified and normalized to those of total ATG16L1. The data are the mean ± SEM, n = 3, ∗∗*p* < 0.01, ∗∗∗*p* < 0.001 *versus* EGF. Statistical analyses were performed using one-way ANOVA with Bonferroni’s multiple comparisons test. EGF, epidermal growth factor; MVK, methyl vinyl ketone.

To investigate the effects of MVK on physiological events, we focused on autophagy. Activated Akt induces the activation of mTOR, which is a part of mTORC1 and is a regulator of cell proliferation and autophagy (49, 50), and activated mTORC1 suppresses unc-51 like autophagy activating kinase 1 activity and autophagosome formation (15). Since EGF attenuates serum starvation-induced autophagy (51), we expected that MVK would reverse the negative regulation of autophagy by EGF. During autophagy induction, LC3 translocates and accumulates on the autophagosome membrane, forming punctate structures. Additionally, ATG16L1 phosphorylation induced by unc-51 like autophagy activating kinase 1 (52) is a marker of autophagosome formation that can be analyzed without considering autophagy flux (53). To investigate the effects of MVK on autophagy, we determined the number of LC3 puncta, markers of mature autophagosomes, and analyzed the phosphorylation of ATG16L1. When we treated serum-starved A549 cells with 20 ng/ml EGF and counted LC3 puncta upon immunostaining using the difference of Gaussians method of ImageJ (https://imagej.net/ij/index.html) (54), we found that the number of LC3 puncta decreased (Fig. 5, *A* and *B*). However, pretreatment with MVK dose dependently restored the number of LC3 puncta (Fig. 5, *A* and *B*), suggesting that MVK abolished the effect of EGF on LC3 punctus formation and induced autophagy. To confirm that this effect of MVK is involved in the suppression of PI3K–Akt signaling, we treated with BYL719, which is a PI3Kα specific inhibitor (55) and found that BYL719 reverse EGF-suppressed effect on the number of LC3 puncta and on ATG16L1 phosphorylation (Figs. 5*C–E* and S4*A*). Additionally, MVK abolished the suppression of ATG16L1 phosphorylation by EGF, and this phosphorylation was not induced by MVK alone (Fig. 5, *D* and *E*). Our data suggested that MVK counteract the effects of EGF on autophagy through suppression of PI3K–Akt signaling.

**Figure 6:**
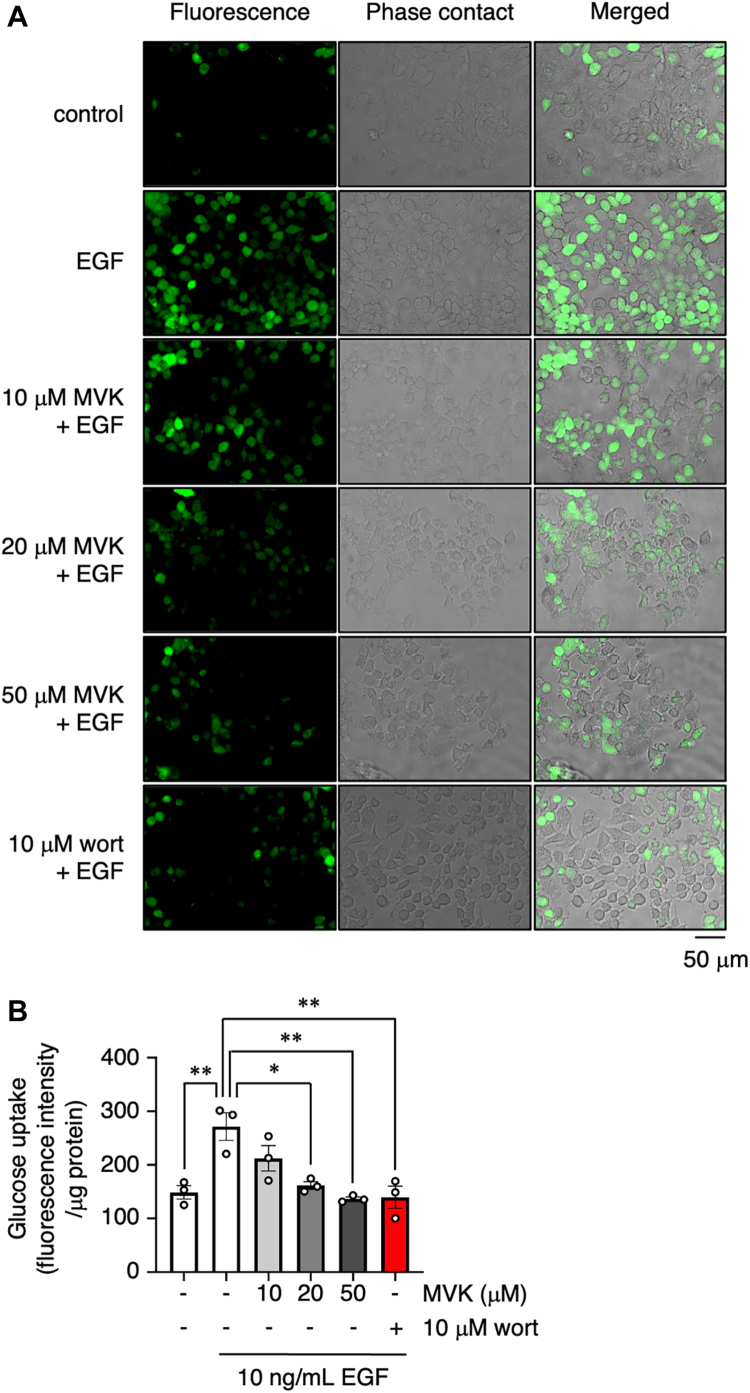
**MVK attenuates EGF-induced glucose uptak**e. *A*, serum-starved A549 cells were treated with the indicated concentration of MVK or 10 μM wort in serum- and glucose-free medium for 30 min. The cells were incubated with 10 ng/ml EGF and Glucose Uptake Probe-Green for 60 min, and phase-contrast and fluorescence images were observed after washing away the probe. The scale bar represents 50 μm. *B*, the fluorescence intensity of the cell lysates was normalized to the concentration of total protein. The data are the mean ± SEM, n = 3, ∗*p* < 0.05, and ∗∗*p* < 0.01 *versus* EGF. Statistical analysis was performed using one-way ANOVA with Bonferroni’s multiple comparisons test. EGF, epidermal growth factor; MVK, methyl vinyl ketone.

Next, to further analyze the effects of MVK on PI3K–Akt signaling–mediated physiological responses, we focused on glucose uptake. Activation of Akt by growth factors or insulin induces glucose uptake through membrane translocation of the glucose transporter including GLUT1 and GLUT4 (16, 17, 18). Interestingly, it has been reported that mutations in the cSH2 domain of p85 cause insulin resistance by suppressing glucose uptake (43). To examine the effect of MVK on glucose uptake, A549 cells were cultured in serum and glucose-free medium and treated with MVK and EGF. The cells were then treated with Glucose Uptake Probe-Green to detect intracellularly incorporated glucose according to the fluorescence intensity. As EGF-induced glucose uptake was inhibited by wort treatment, it was also inhibited by MVK in a dose-dependent manner (Fig. 6, *A* and *B*).

Physiological phenomena regulated by independent RTKs, namely, autophagy and glucose uptake, were both regulated by MVK, suggesting that MVK regulates physiological mechanisms through PI3K–Akt signaling, a common regulatory pathway.

### MVK structural analogs suppress the phosphorylation of Akt

**Figure 7:**
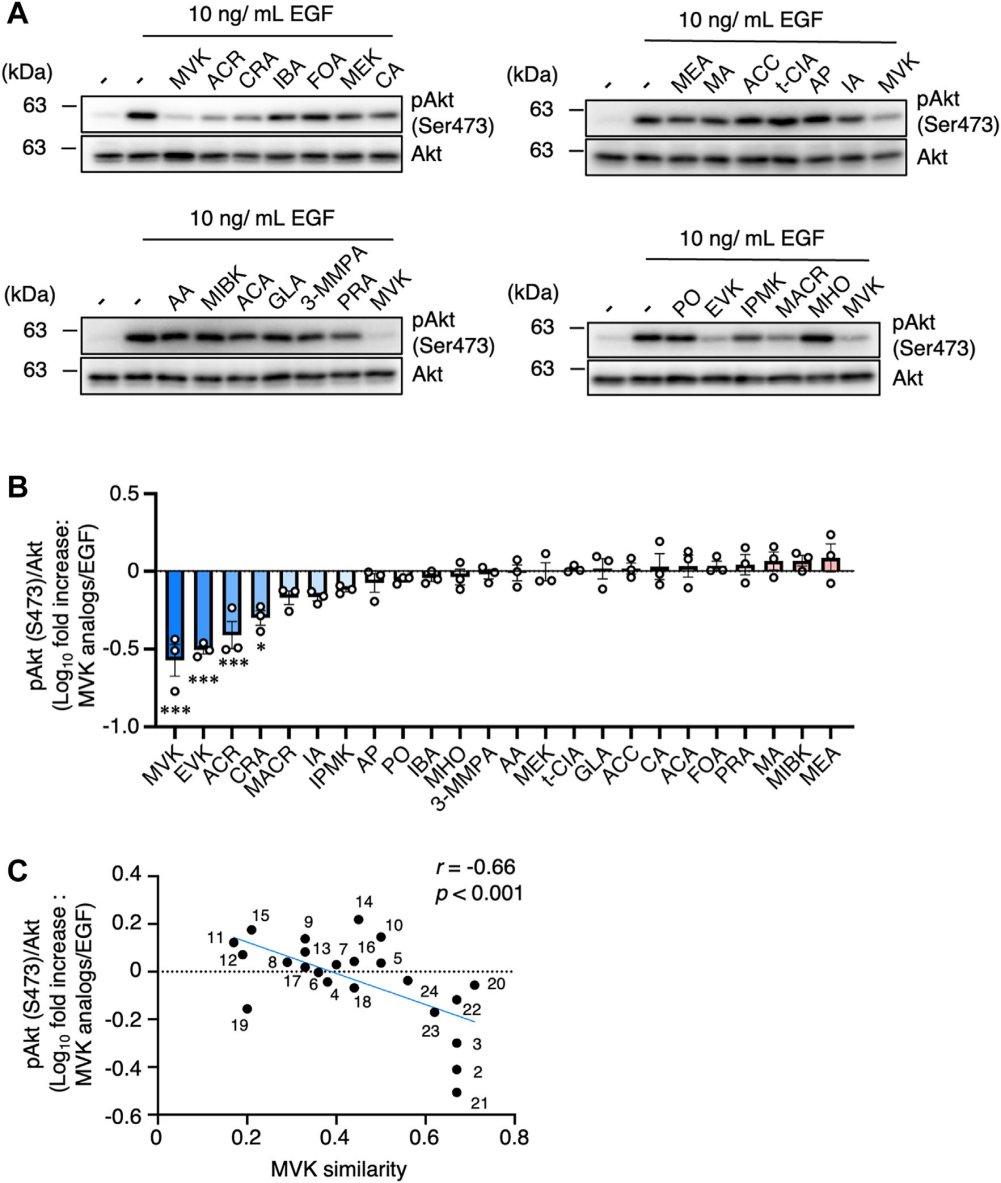
**MVK analogs tend to suppress Akt phosphorylation in correlation with MVK similarity**. *A*, A549 cells were incubated with serum-free medium for 24 h and exposed to 50 μM MVK analogs for 30 min. After stimulation with 10 ng/ml EGF for 10 min, the lysates were analyzed by Western blotting with anti-pAkt and Akt antibodies. *B*, the levels of pAkt were quantified and normalized to that of total Akt. The ratio of the log10-transformed pAkt/Akt value of each RCS-treated sample to that of the EGF-treated sample was calculated, and the resulting ratios are ranked in descending order. The data are the mean ± SEM, n = 3, ∗*p* < 0.05, ∗∗*p* < 0.01, and ∗∗∗*p* < 0.001 *versus* EGF. Statistical analysis was performed using one-way ANOVA with Bonferroni’s multiple comparisons test. *C*, the correlation between MVK similarity and suppression of Akt phosphorylation was analyzed using linear regression analysis in GraphPad Prism 8. EGF, epidermal growth factor; MVK, methyl vinyl ketone; RCS, reactive carbonyl species.

We examined whether RCS with a high structural similarity to MVK also suppress PI3K–Akt signaling. To evaluate structural similarity to MVK in silico, the chemical structures of MVK and compounds were hashed with an algorithm using the MACCS key fingerprint (56), and the Tanimoto coefficient, which is commonly used to quantify molecular similarity (57), between MVK and each compound was calculated in RDKit (58). We selected 23 representative compounds among RCS (Table S1) based on the following criteria: (1) a low molecular weight, (2) high abundance in the environment, and (3) a Tanimoto coefficient higher than 0.15. We then analyzed the effects of each analog on Akt phosphorylation. Serum-starved A549 cells were pretreated with 50 μM MVK analogs (the maximum concentration used for MVK) for 30 min and stimulated with EGF. The results showed that ACR and CRA with an α,β-unsaturated aldehyde moiety markedly suppressed Akt phosphorylation as effectively as MVK (Figs. 7, *A* and *B*, S5, and Table S1). These results are consistent with previous findings that ACR and CRA negatively regulate the PI3K–Akt pathway (59, 60, 61) and may target p85 as effectively as MVK. Among chemicals with an α,β-unsaturated carbonyl, EVK, which has one more C chain, showed almost the same effect as MVK, and itaconic acid, isopropenyl methyl ketone, and methacrolein had a weak inhibitory effect on Akt phosphorylation (Figs. 7 *A* and *B*, S5, and Table S1).

Interestingly, not all MVK analogs suppressed Akt phosphorylation. Methyl ethyl ketone, which has the same structure as MVK without C–C double bond was no repression of Akt phosphorylation (Figs. 7, *A* and *B*, S5, and Table S1). Similar results were also seen in the chemicals without α,β-unsaturated carbonyl group such as isobutylaldehyde, formaldehyde, methyl isobutyl ketone, acetaldehyde, glutaraldehyde (GLA), 3-(methylmercapto), and propionaldehyde (Fig. 7, *A* and *B* and Table S1). These results suggest that the α,β-unsaturated carbonyl group plays an important role in suppressing Akt phosphorylation. However, among chemicals with an α,β-unsaturated carbonyl, crotonic acid, AA, acryloyl chloride (ACC), t-CIA, acetophenone (AP), PO, and 5-methyl-3-hexen-2-one are without effects on inhibition of Akt phosphorylation (Fig. 7 *A* and *B* and Table S1).

In addition, we analyzed the correlation between MVK similarity and suppression of Akt phosphorylation using linear regression analysis in GraphPad Prism 8 (https://www.graphpad.com/). We found a positive correlation between structural similarity to MVK and suppression of Akt phosphorylation (Fig. 7*C*). Although other factors are expected to intervene, we showed that MVK similarity is an important factor in assessing the effects of compounds on the suppression of PI3K–Akt signaling.

## Discussion

It is widely recognized that various chemicals in the environment are deeply involved in human health. However, most analyses of these compounds are based on indirect toxicity assessments, such as cell death assessment. Analytical models are needed to better understand the toxicity of environmental factors.

In this study, we investigated whether environmental RCS affect the activity and function of PI3K–Akt signaling, which regulates homeostatic mechanisms in living organisms. We found that MVK, one of the RCS, inhibits the interaction between RTKs and PI3K, and the modification site of PI3K p85 subunit by MVK is important for this interaction (Figs. 2 and 3), but further studies are needed to demonstrate that MVK binding to Cys656 of the PI3K p85 directly suppresses PI3K–Akt signaling. We also found that MVK negatively regulates PI3K–Akt signaling (Figs. 1 and 4) and impairs physiological functions such as autophagy and glucose uptake (Figs. 5 and 6). Known PI3K inhibitors such as wortmannin and LY294002 bind to the ATP pocket of the p110 subunit and inhibit p110 kinase activity (62), but our studies with MVK suggest a novel mode of PI3K inhibition targeting p85.

Furthermore, our study suggests that detailed modeling of one representative RCS may enable extension of the analysis to RCS analogs. Interestingly, among the 23 RCS compounds with structures similar to that of MVK, ACR, CRA, and EVK suppressed Akt phosphorylation, suggesting that these analogs share a common physiological mechanism with MVK. Interestingly, α,β-unsaturated carbonyls, which suppress Akt phosphorylation, were limited (Fig. 7). A reasonable explanation is that a carbon atom at the β-position of crotonic acid, t-CIA, AP, PO, and 5-methyl-3-hexen-2-one are alkylated, and thus steric hindrance might occur to access and bind to Cys residue of p85. In addition, ACC is easily decomposed in water to form acrylate, which is a weak electrophile as well as AA (63). Although this study could not reveal an obvious structure-activity relationship between Akt phosphorylation inhibition and MVK-related chemicals, our study elucidates the detailed molecular mechanisms of the toxicity of MVK and its analogs and provides an effective model for the assessment of environmental reactive species.

As previously reported by proteomic analysis of ACR (64), we expected that MVK affects not only PI3K–Akt signaling but also on other proteins. To fully understand the effects of MVK, we need to further analysis on other signaling, including mitogen-activated protein kinase–extracellular signal-regulated kinase signaling.

The smoke of one cigarette contains approximately 30 μg of MVK (22). Additionally, when the gas phase of ten cigarettes was collected and dissolved in 10 ml of water, the concentration of MVK was approximately 2500 μM (65). We consider that the maximum concentration (50 μM) of MVK and MVK analogs used in this study is within the range of concentrations that can be expected from human exposure to cigarette smoke.

Although there are few reports of *in vivo* studies on MVK, it has been reported that MVK accumulates in the liver and kidney and exhibits cytotoxicity there (25, 66). In addition, ACR, which has structural similarities to MVK, is absorbed systemically after oral or inhalation exposure in rats (67), and high blood levels of ACR have been reported in smokers (68). Therefore, we expect that the effects of ACR in addition to MVK on PI3K–Akt signaling are not specific to the lungs.

It is well known that activation of the PI3K–3-phosphoinositide-dependent protein kinase-1 (PDK1) pathway induces activation of Akt, whereas the PI3K–mTORC2 pathway also induces Akt phosphorylation at Ser473 (29). In this study, MVK suppressed the phosphorylation of Akt at Thr308, which is a target site of PDK1 (Fig. 1), suggesting that the PI3K–PDK1–Akt pathway plays an important role in the effect of MVK. However, since the contribution of the PI3K–mTORC2–Akt pathway is also highly conceivable, a detailed analysis of the effects of MVK on these pathways is required.

Our results indicate that MVK binds to Cys656 of p85 (Fig. 3). In addition to the recruitment and stabilization of p110, p85 contributes to RTK recognition and binding (37). Although Cys656 of p85 is highly conserved across animal species and PI3K isoforms (Fig. S5), the function and posttranslational modifications of Cys656 have not been reported. We show here for the first time that Cys656 is responsible for the binding of p85 to RTKs (Fig. 3, *B* and *C*). Although the detailed binding sites of EGFR to p85 are controversial, it has been hypothesized that Tyr920, part of a YXXM motif in the kinase domain of EGFR, interacts with the SH2 domain in p85 (69, 70, 71) and is considered at least a critical domain for mutual binding. MVK did not bind to the iSH2 domain (Table S2), which interacts with p110 (38), suggesting that MVK has little effect on the p85–p110 interaction. Furthermore, MVK did not bind to any nucleophilic residues (Lys, His, etc.) other than Cys, suggesting that MVK selectively binds to Cys residues (Table S2). In particular, Cys656 is exposed on the protein surface, unlike other Cys residues in the cSH2 domain, such as Cys659 and Cys670 (41) (Fig. S3*B*). Differences in the accessibility of Cys residues due to the PI3K conformation are expected to influence the sensitivity to MVK modification.

In this study, we focused on autophagy and glucose uptake among the physiological functions regulated by PI3K–Akt signaling (Figs. 5 and 6). Generally, activation of autophagy participates in the removal of dysfunctional proteins as a cytoprotective effect. However, excessive activation of autophagy has been reported to cause autophagy-induced cell death and various diseases, such as neurodegenerative diseases, suggesting the importance of maintaining autophagy homeostasis (72).Therefore, MVK exposure is thought to disrupt autophagy homeostasis and lead to adverse physiological effects. We also showed that MVK attenuates glucose uptake into the cell (Fig. 6). Previous reports have shown that impaired glucose uptake causes insulin resistance and that high blood glucose levels are involved in diabetes and diabetic complications such as neuropathy, renal damage, and obesity (73, 74). Interestingly, cigarette smoke extract containing MVK and its related chemicals with α,β-unsaturated carbonyl (75) is reported to reduce glucose uptake and Akt activation (76) and causes growth failure and diabetes (20, 21). We therefore speculate that RCS like MVK may, at least in part, contribute to the effect caused by cigarette smoke extract.

In previous reports, PI3K p85 with mutations in the cSH2 domain have been shown to attenuate PI3K–Akt signaling *via* defective binding to RTKs and to cause SHORT syndrome, which is characterized mainly by growth and developmental failure, abnormal glucose metabolism, and diabetes (42, 43). Our study suggests that MVK may cause SHORT syndrome-like symptoms such as growth failure and diabetes mellitus through attenuation of suppression of cell proliferation, suppression of autophagy, and glucose uptake *via* regulation of PI3K–Akt signaling. In addition, our MVK model of p85 dysfunction may serve as a useful model for SHORT syndrome and related disorders and could be used to develop treatments for these conditions.

Although there are countless RCS, our results enable efficient evaluations of the effects of RCS on PI3K–Akt signaling *via* consideration of the similarity to the indicator MVK. In addition, it is assumed that combined exposure to MVK and MVK analogs shows cumulative effects on the suppression of PI3K–Akt signaling (Fig. S6). Interestingly, RCS found in not only the atmosphere, such as MVK, ACR, and CRA, but also, in food additives for flavor, such as EVK, suppressed Akt phosphorylation (Fig. 7). Our study suggests that MVK and its analogs can affect PI3K–Akt signaling *in vivo* through diverse exposure pathways.

Exposome was defined as the group of complex and cumulative effects of various chemicals in the living environment, diet, and endogenous processes on biological responses over a lifetime (77). In particular, electrophilic environmental chemicals should be given priority for evaluation not only because they are routinely and chronically exposed in daily life but also because they form protein adducts and disrupt the functions of proteins (78, 79). We previously reported that 1,2-naphthoquinone, which is found in air pollutants such as particulate matter 2.5, activates EGFR *via N*-arylation and induces PI3K–Akt signaling (80). Our studies indicate that the disruption of signaling homeostasis by 1,2-naphthoquinone, MVK, and MVK analogs leads to adverse physiological effects and demonstrate the importance of protein adduct formation by environmental electrophiles as a model for exposome research. Although there are several issues that need to be resolved, including a need for assessment of the pathophysiological mechanisms of environmental electrophiles, a detailed elucidation of the effects of individual RCS, as in this study, is the first step toward considering the effects of environmental factors as a whole and may help lead to the development of methods for the prevention and treatment of related diseases.

## Experimental procedures

### Reagents and antibodies

The RCS are listed in Table S1.

Antibodies against Akt (#9272S), phospho-Akt (Ser473) (D9E) (#4060), phospho-Akt (Thr308) (D25E6) (#13038), EGFR (D38B1) (#4267), phospho-EGFR (Tyr1068) (D7A5) (#3777), IR (4B8) (#3025), phospho-IR (Tyr1150/1151) (#3021), Bad (D24A9) (#9239), phospho-Bad (Ser136) (D25H8) (#4366), phospho-GSK3β (Ser9) (D85E12) (#5558), β-actin (13E5) (#5125), phospho-ATG16L1 (Ser278) (#84300), and ATG16L1 (D6D5) (#8089) were purchased from Cell Signaling Technology. Antibodies against PI3K (610045) and GSK3β (610201) were purchased from BD Transduction Laboratories. Antibodies against phospho-PI3K p85a (Tyr607) (ab182651) and SC-79 (ab146428) were purchased from Abcam. Antibodies against FLAG (DYKDDDDK) (A8592) were purchased from Sigma–Aldrich. Antibodies against LC3 (PM036) were purchased from Medical & Biological Laboratories. The following antibodies were purchased from GE Healthcare: anti-mouse IgG-horseradish peroxidase (NA9310) and anti-rabbit IgG-horseradish peroxidase (NA9340). Recombinant human EGF (236-EG) was purchased from R&D Systems. Recombinant human insulin (096-03443), sodium orthovanadate, and wort were purchased from Fujifilm–Wako. Recombinant human PI3K (p110a/p85a) (V1721) was purchased from Promega. BYL719 (S2814) was purchased from Selleck.

### DNA construction and mutagenesis

The expression vector for FLAG-tagged WT EGFR (pIDT-SMART [C-TSC] EGFR-FLAG) was prepared as described previously (81). The complementary DNA encoding the PI3K p85 protein was derived from the Genome Network Project (RIKEN BRC) clone and amplified by Tks Gflex DNA Polymerase (TaKaRa Bio, R060A) using the following primer pair: 5′-CCC CGG ATC CCC ACC ATG TAC AAT ACT GTT TGG AAT-3′ and 5′-GGG GAG CGG CCG CCA TCG CCT CTG CTG TGC ATA-3’. The resulting PCR product was digested with *Bam* HI-HF (New England Biolabs, R3136S) and *Not*I (New England Biolabs, R0189S), and subcloned into the similarly digested pcDNA6 myc-His vector (Thermo Fisher Scientific, V22120).

PCR-based site-directed mutagenesis was performed using PrimeSTAR Max DNA polymerase (Takara Bio, R046A) using the following primer pairs: C146S PI3K p85 forward, 5′-CTG GAA TCT TCA ACT CTA TAC AGA ACA-3′, C146S PI3K p85 reverse, 5′-CTT TTC TTT CCA GAC CTT AGA AGT TGA-3’; C656S PI3K p85 forward, 5′- CAG GGC TCC TAT GCC TGC TCT GTA GTG-3′, and C656S PI3K p85 reverse, 5′- GGC ATA GGA GCC CTG TTT ACT GCT CTC-3’.

The inserted and mutated sequences were confirmed by Sanger sequencing (GENEWIZ).

### Cell culture and transfection

A549 human lung adenocarcinoma epithelial cells and MDA-MB-231 human breast adenocarcinoma cells were cultured in Dulbecco’s modified Eagle’s medium supplemented with 10% (v/v) heat-inactivated fetal bovine serum at 37 °C in a humidified atmosphere of CO_2_/95% air. Human small airway epithelial cells were cultured in SABM Basal Medium (CC-3119) with SAGMTM SingleQuots Supplement Pack (CC-4124) at 37 °C in a humidified atmosphere of CO_2_/95% air. Transfections were conducted using PEI MAX (Polysciences, 2465-1) according to the manufacturer’s instructions.

### Western blotting

Cells were washed with PBS and lysed in ice-cold radioimmunoprecipitation assay buffer (50 mM Tris–HCl [pH 7.5], 150 mM NaCl, 1% [w/v] sodium deoxycholate, 0.1% [w/v] SDS, 1% [v/v] Triton X-100, and a protease inhibitor cocktail) with PhosSTOP (a phosphatase inhibitor cocktail, Roche Diagnostics, 4906845001) on ice for 5 min. After quantification of protein concentration by the bicinchoninic acid (BCA) assay (Takara, T930A) method, protein samples were boiled in 1×Laemmli SDS sample buffer (62.5 mM Tris–HCl [pH 6.8], 5% [v/v] 2-mercaptoethanol, 2% [w/v] SDS, and 10% [v/v] glycerol) for 5 min. The samples were subjected to SDS–PAGE and Western blotting as previously described (80).

### pNPP phosphatase assay

Cells were washed with PBS and lysed in ice-cold cell lysis buffer (50 mM Tris–HCl [pH 7.5], 150 mM NaCl, 1% Nonidet P-40 [NP-40], and a protease inhibitor cocktail) on ice for 5 min. The total protein concentrations were measured by BCA protein assay. Cell lysates (150 μg) were incubated with 10 mM pNPP (New England Biolabs, P0757S) and 0.1 mg/ml bovine serum albumin at 37 °C for 10 min. The reaction was terminated under alkaline conditions by incubation with 0.9 M NaOH. The formed pNP was quantified 405 nm using a microplate reader (iMark, Bio-Rad).

### PI3K activity assay

A PI3K activity assay was conducted using a PI3K Activity ELISA: Pico Kit (Echelon Biosciences, K-1000s) according to the manufacturer’s instructions with a slight modification. Briefly, recombinant human PI3K (p110a/p85a) (20 ng/ml) was incubated with MVK or 1 μM wort for 30 min at 37 °C. PI3K was incubated with 5 μM PIP2 substrate for 3 h at 37 °C. The reaction was terminated by the addition of STOP buffer. Colorimetric ELISA analysis of PIP3 levels was performed by measuring the *A*_450_ using a microplate reader (iMark, Bio-Rad).

### Coimmunoprecipitation

Cells were washed with PBS and lysed in ice-cold NP-40 buffer (50 mM Tris–HCl [pH 7.5], 150 mM NaCl, 1% NP-40, 5 mM EDTA, and a protease inhibitor cocktail) with PhosSTOP on ice for 5 min. After quantification of the protein concentration by BCA protein assay, the cell lysates were incubated first with an anti-FLAG (M2) monoclonal antibody for 2 h at 4 °C and then with protein G Sepharose beads (GE Healthcare, 17061801) for 2 h at 4 °C. After washing with NP-40 buffer, the samples were eluted from the beads with 1 × Laemmli SDS sample buffer.

### LC–MS/MS assay

Recombinant PI3K (10 μg/ml) was incubated with 20 μM MVK for 10 min at room temperature (RT). The protein mixture was reduced and carboxymethylated to remove the S–S bond. After SDS–PAGE and Coomassie brilliant blue staining, the bands were excised and digested using trypsin or AspN. The digestion mixture was subjected to Q Exactive mass spectrometry (MS). MS and tandem MS data were acquired using the data-dependent top10 method. The resulting tandem MS data were searched by MASCOT 2.7 (https://www.matrixscience.com/) and quantified using Proteome Discoverer2.4 (https://www.thermofisher.com/jp/en/home/industrial/mass-spectrometry/liquid-chromatography-mass-spectrometry-lc-ms/lc-ms-software/multi-omics-data-analysis/proteome-discoverer-software.html) with variable modifications. In Table S2, the MVK modification site is indicated as a CRA modification because CRA modification is registered in the database with the exact same composition as MVK modification. The residues that were more than 1% bound to MVK compared with the control were designated MVK target residues (Table S3).

### LC3 puncta detection

A549 cells were cultured on coverslips and incubated with serum or serum-free medium for 24 h. After exposure to MVK for 30 min or BYL719 for 60 min, the cells were incubated with 20 ng/ml EGF for 60 min. Then, the cells were washed two times with PBS, fixed with 10% (v/v) paraformaldehyde in PBS for 20 min at RT, washed three times, and permeabilized with PBS-Triton-X (0.1% [v/v]) for 10 min at 4 °C. The cells were then blocked with 1% (w/v) bovine serum albumin in PBS for 1 h at RT and incubated with a primary anti-LC3 antibody (1:500) overnight at 4 °C. The cells were washed three times with PBS and were then incubated with an Alexa 594–conjugated goat anti-rabbit IgG antibody (1:400) (Invitrogen, A-11005) for 30 min at RT. Coverslips were mounted with VECTASHIELD with 4′,6-diamidino-2-phenylindole (Vector Laboratories, Inc.) overnight at RT, and fluorescence was examined using a fluorescence microscope (BZ-X800, Keyence). The LC3 puncta were counted using the difference of Gaussians method of ImageJ (https://imagej.net/ij/index.html) (54) in three fields and normalized to the number of cells in three independent experiments.

### Glucose uptake assay

A glucose uptake assay was conducted using a Glucose Uptake Kit-Green (Dojindo, UP02) according to the manufacturer’s instructions. A549 cells were serum-starved for 24 h. The cells were washed with serum- and glucose-free medium and incubated at 37 °C for 30 min. EGF (10 ng/ml) and Glucose Uptake Probe-Green (diluted 500 times with PBS) were added after 30 min of MVK treatment; the cells were incubated for 60 min and then washed in ice-cold wash buffer. The cells were immediately observed by phase-contrast and fluorescence microscopy (Ex: 490 nm, Em: 530 nm) (BZ-X800, Keyence). After imaging, the cells were lysed in ice-cold radioimmunoprecipitation assay buffer. The cell lysates were transferred into a 96-well black plate, and fluorescence intensity (Ex: 475 nm, Em: 500–550 nm) was examined using a fluorescence microplate reader (GloMax Explorer Multimode Microplate Reader, Promega). The total protein concentrations were measured by BCA protein assay.

### Calculation of MVK similarity

The chemical structures of MVK and 23 MVK analogs were hashed with MACCS key fingerprints (56) in RDKit (57). Then, the Tanimoto coefficient between MVK and each analog was calculated in RDKit. The calculated Tanimoto coefficient is expressed as MVK similarity.

### Statistical analysis

All experiments were independently performed at least three times. All data are expressed as the mean ± SEM values. The experiments were analyzed using one-way ANOVA with Bonferroni’s multiple comparison test using GraphPad Prism 8 (GraphPad Software, https://www.graphpad.com/). *p* values of <0.05 were considered to indicate significant differences.

## Data availability

All data are contained within the article and the Supporting information.

## Supporting information

This article contains Supporting information.

## Conflict of interest

The authors declare that they have no conflicts of interest with the contents of this article.
