# Supplementary material for: Methyl vinyl ketone and its analogs covalently modify PI3K and alter physiological functions by inhibiting PI3K signaling
Source: J Biol Chem. 2024 Jan 24;300(3):105679. doi: 10.1016/j.jbc.2024.105679 (PMC10881440; doi:10.1016/j.jbc.2024.105679)
Supplement: Supporting figures [file mmc1.docx]

Supporting Information

Methyl vinyl ketone and its analogues covalently modify PI3K and alter physiological functions by inhibiting PI3K signaling

Atsushi Morimoto^1^, Nobumasa Takasugi^1^, Yuexuan Pan^1^, Sho Kubota^1^, Naoshi Dohmae^2^, Yumi Abiko^3^, Koji Uchida^4^, Yoshito Kumagai^5^, Takashi Uehara^1, *^

^1^*Department of Medicinal Pharmacology, Graduate School of Medicine, Dentistry and Pharmaceutical Sciences, Okayama University, Okayama, Japan*

^2^*Biomolecular Characterization Unit, Technology Platform Division, RIKEN Center for Sustainable Resource Science, Wako, Saitama, Japan*

^3^*Graduate School of Biomedical Science, Nagasaki University, Nagasaki, Japan*

^4^*Laboratory of Food Chemistry, Graduate School of Agricultural and Life Sciences, The University of Tokyo, Tokyo, Japan*

^5^*Graduate School of Pharmaceutical Sciences, Kyushu University, Fukuoka, Japan*

*To whom correspondence should be addressed: Takashi Uehara, Ph.D., email: uehara-t@okayamau.ac.jp;

**File Name:** Supporting Figures

**Description:** 7 Supporting Figures


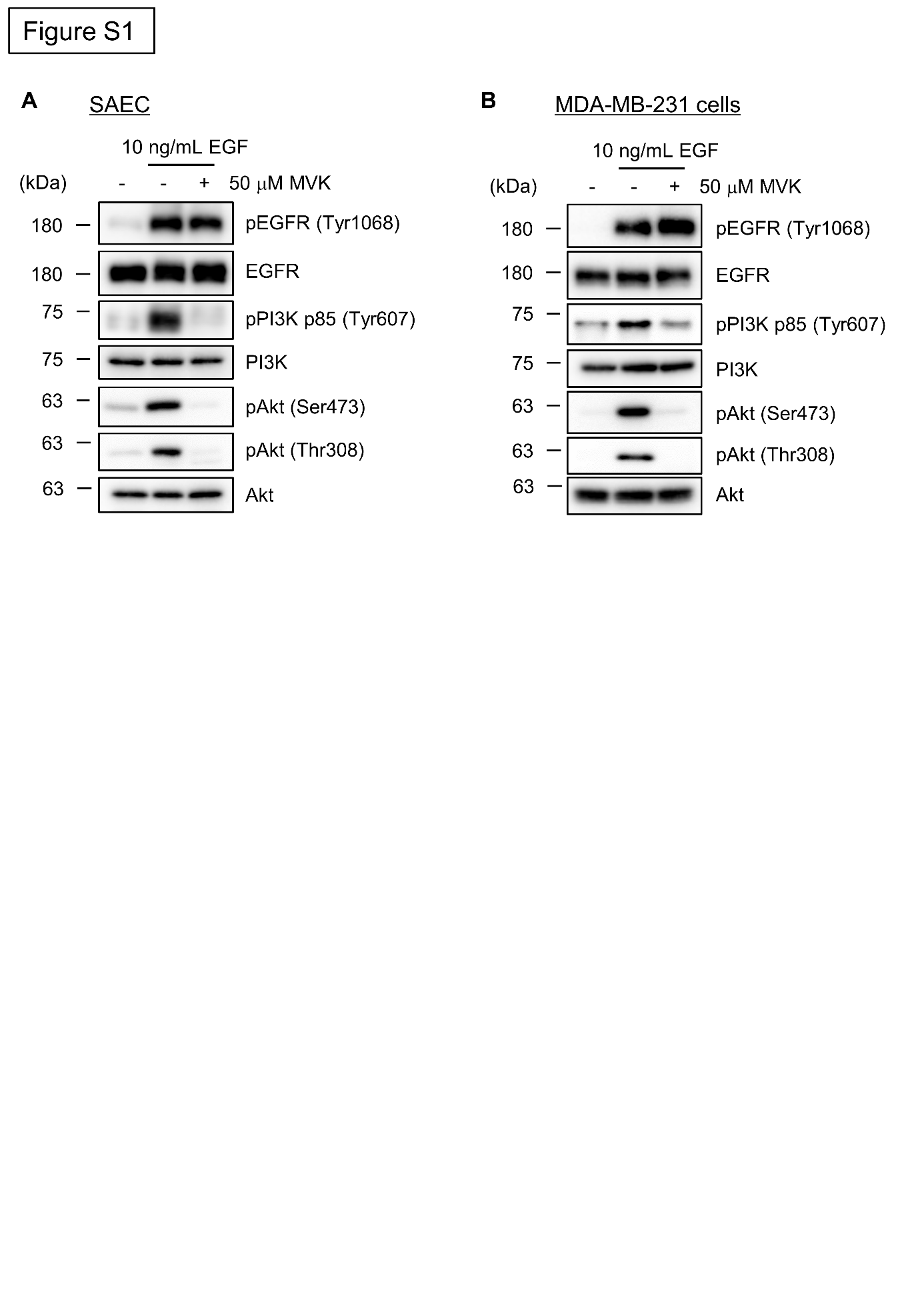


**Figure S1 The effect of MVK on EGFR–PI3K–Akt pathway in other cell lines**

*A* and *B*, Human small airway epithelial cells were incubated with only SABM^TM^ Basal Medium for 4 h (A) or MDA-MB-231 cells were incubated with serum-free medium for 24 h (B) at 37 °C. After exposure to 50 µM MVK for 30 min, cells were stimulated with 10 ng/mL EGF for 10 min. The lysates were analyzed by Western blotting with anti-pEGFR, anti-EGFR, anti-pPI3K, anti-PI3K, anti-pAkt (Ser473 and Thr308) and anti-Akt antibodies.

**
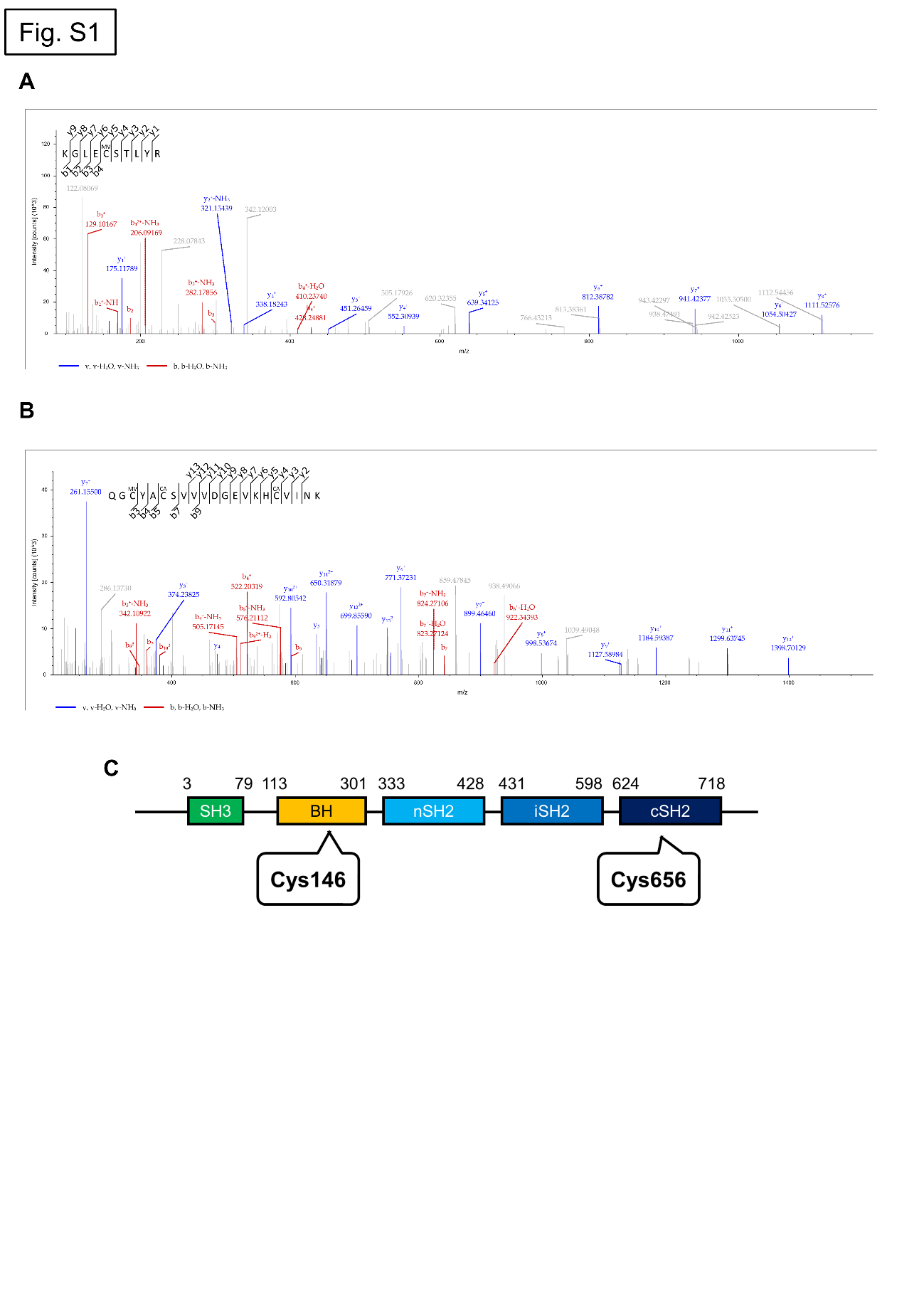
**

**Figure S2 Identification of PI3K p85 modification site by MVK**

*A* and *B*, LC-MS/MS spectra of Cys146 (A) and Cys656 (B) containing peptides. 10 µg/mL recombinant PI3K protein was incubated with 20 µM MVK for 10 min at RT. Trypsin-digested PI3K peptides were analyzed by LC-MS/MS. MV and CA indicate MVK modification and carboxymethylation of Cys residues, respectively. *C*, Schematic diagram of the domain within PI3K p85. p85 consists of five domains: Src homology 3 (SH3) domain, Bcl-2 homology (BH) domain, N-terminal SH2 (nSH2) domain, inter SH2 (iSH2) domain, C-terminal SH2 (cSH2) domain.


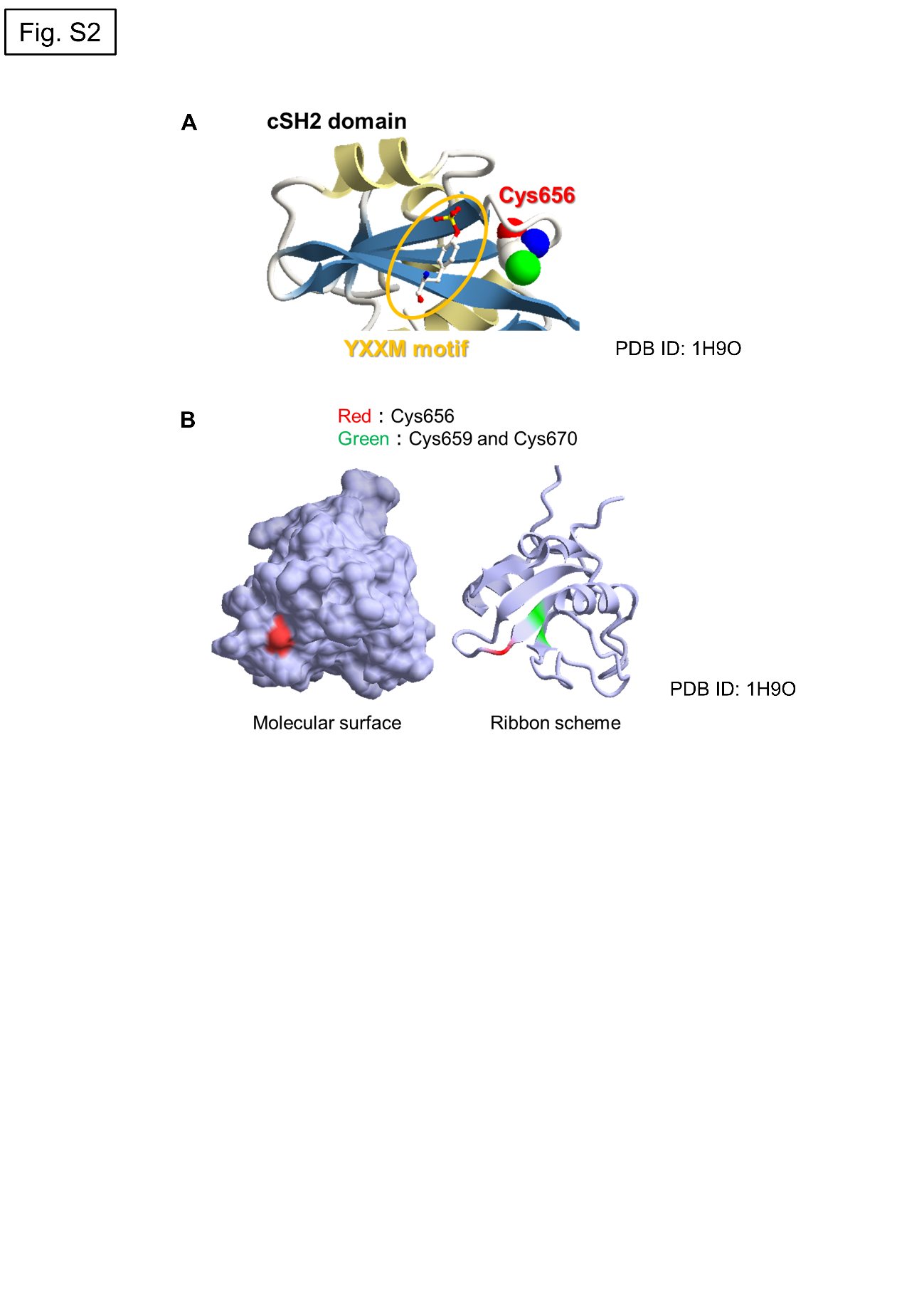


**Figure S3 3-D structure of the cSH2 domain of PI3K p85**

*A*, The crystal structure of the cSH2 domain of p85 in complex with the YXXM motif indicated by the yellow circle (PDB ID: 1H9O). Cys656, which is a target residue of MVK, is shown as a CPK model. *B*, The crystal structure of the cSH2 domain shown in the molecular surface model (left) and the ribbon model (right). The cSH2 domain of p85 consists of three Cys residues: Cys656 (red), Cys659, and Cys670 (green).


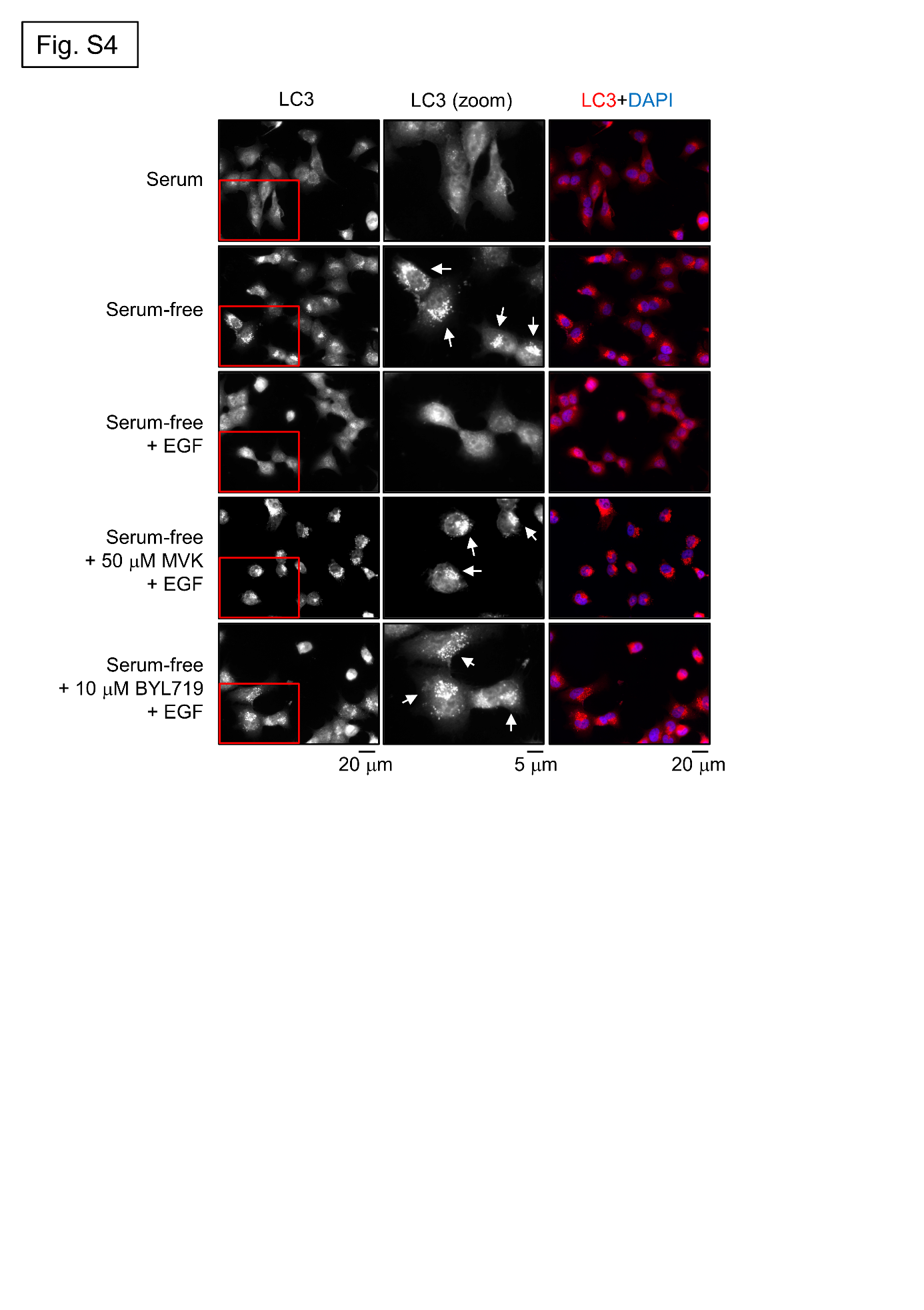


**Figure S4 Effects of MVK or BYL719 on LC3 puncta formation suppressed by EGF**

A549 cells were incubated with serum or serum-free medium for 24 h and exposed to 50 µM MVK for 30 min or 10 µM BYL719 for 60 min. After stimulation with 20 ng/mL EGF for 1 h, the cells were immunostained for LC3 (gray or red) and nuclei (blue). The middle column of panels shows magnified regions indicated by the red frame in the corresponding LC3 images. The arrows indicate LC3 puncta-positive cells. The scale bar represents 20 µm (right and left columns) or 5 µm (middle column).


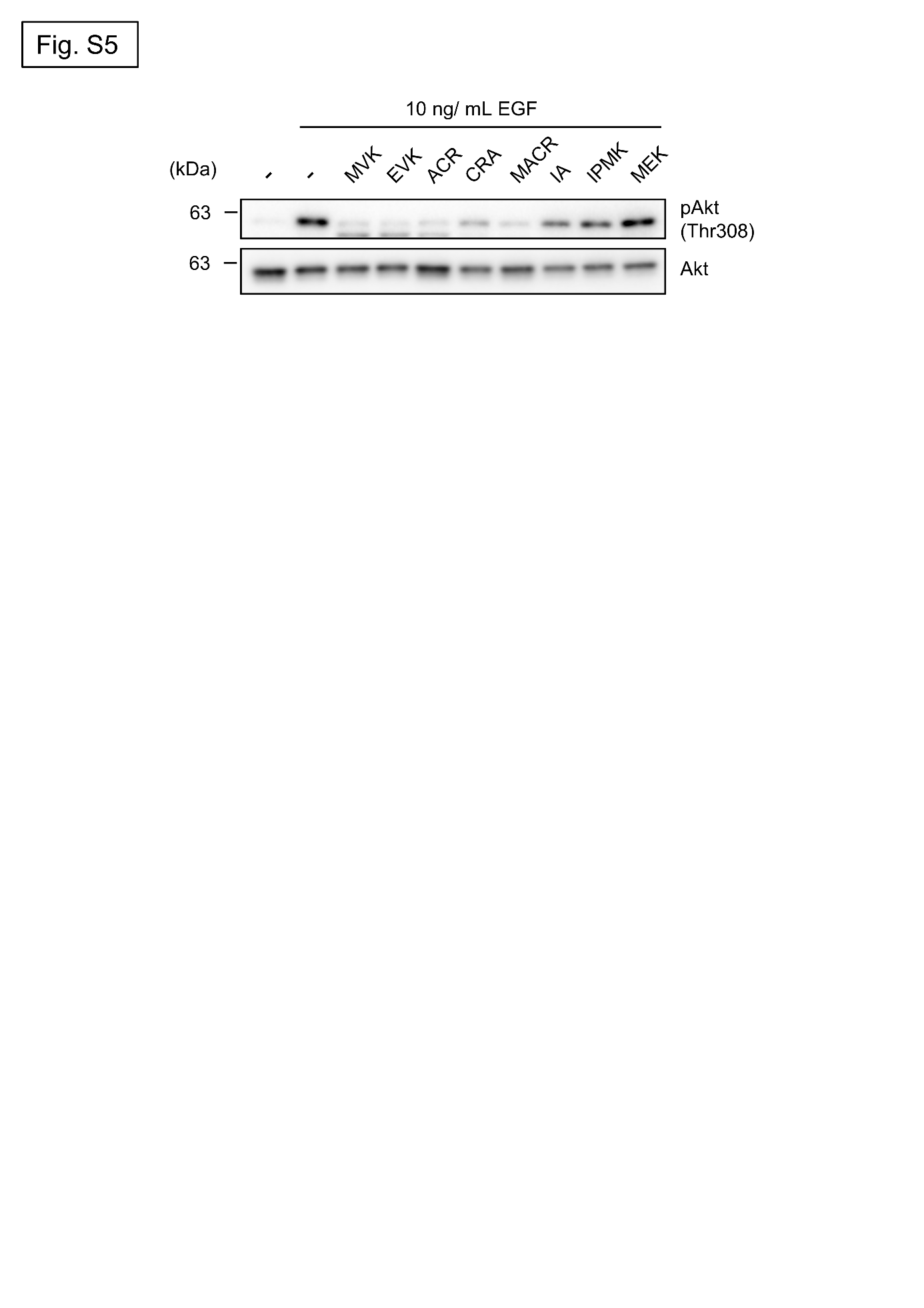


**Figure S5 Effects of** **EVK, ACR, CRA, MACR, IA, IPMK and MEK on the phosphorylation of Akt at Thr308**

A, A549 cells were incubated with serum-free medium for 24 h and exposed to 50 µM MVK, or EVK, ACR, CRA, MACR, IA, IPMK or MEK for 30 min. After stimulation with 10 ng/mL EGF for 10 min, the lysates were analyzed by Western blotting with anti-pAkt (Thr308) and Akt antibodies.


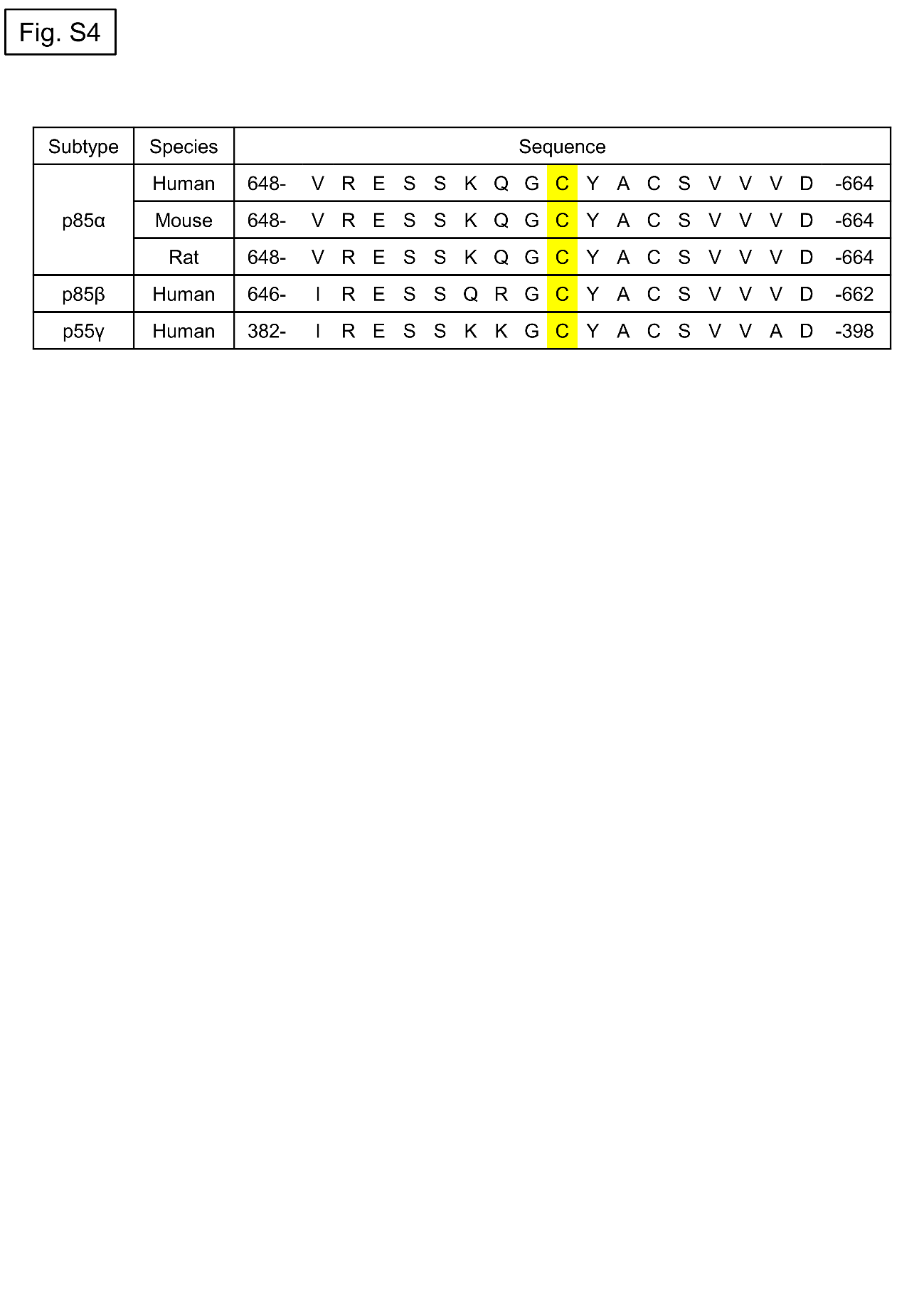


**Figure S6 Conservation of the target Cys of MVK in p85 across isoforms and animal species**

Amino acid sequence alignment around the target Cys of MVK in PI3K p85 across isoforms (p85α, p85β, p55γ) and animal species (human, mouse, rat). The Cys highlighted in yellow indicates the sites of MVK modification in p85.


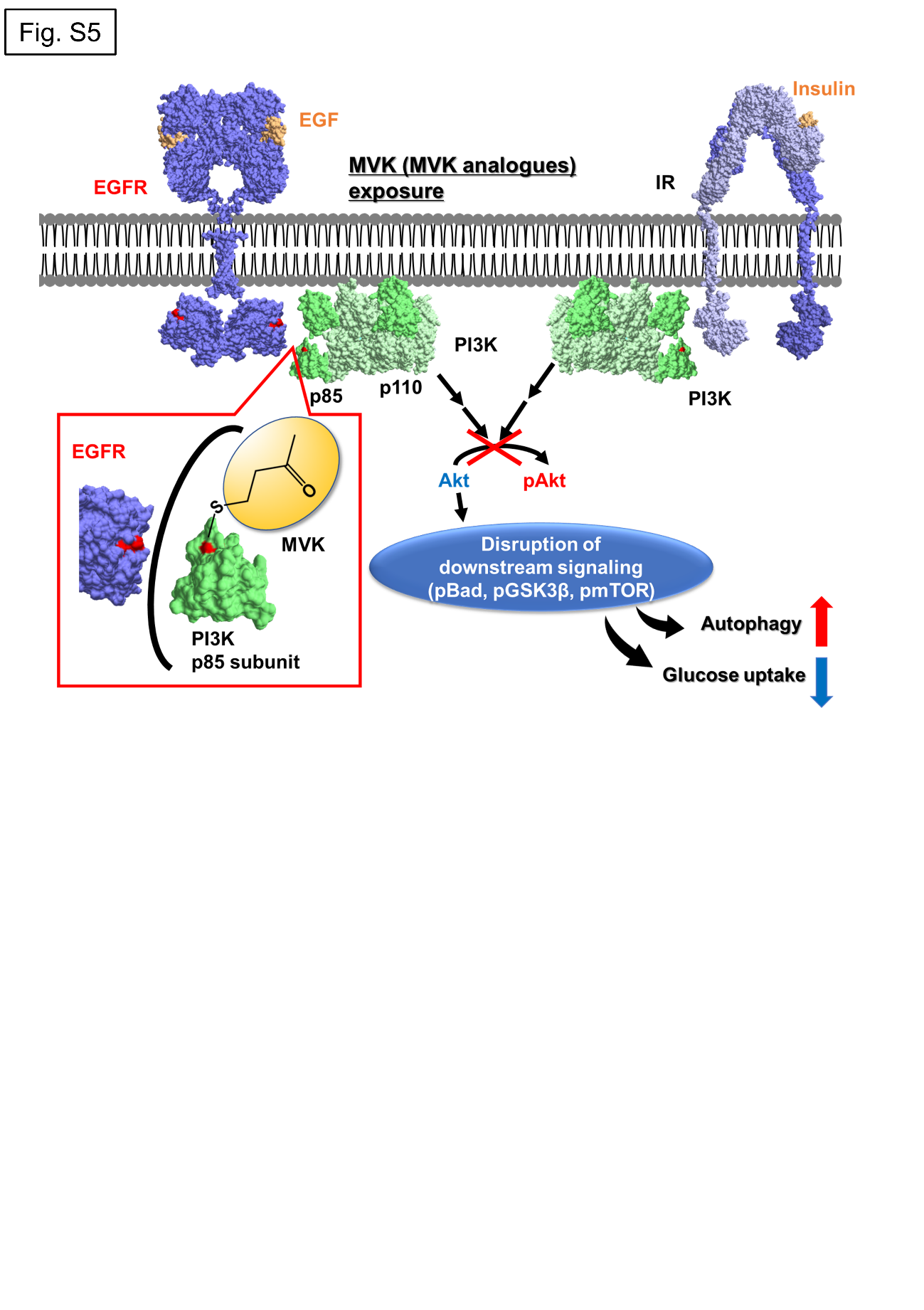


**Figure S7 Graphical abstract**
